# Delayed Primary Repair of Complex Duodenal Injury Associated to Multiorgan Failure Due to Blunt Abdominal Trauma

**DOI:** 10.1055/s-0043-1774404

**Published:** 2023-09-11

**Authors:** José Miguel Aceves-Ayala, Allan Josué Noriega-Velásquez, Alberto Briceño-Fuentes, Cesar Alberto Ortiz-Orozco, Pablo Francisco Rojas-Solís, Pedro Xavier Rivas-Quezada, Carlos Alfredo Bautista-López

**Affiliations:** 1 Surgery Department, Hospital Civil de Guadalajara “Dr. Juan I. Menchaca”, Guadalajara, México; 2 Department of Surgical Clinics, CUCS, University of Guadalajara, Guadalajara, México

**Keywords:** duodenal trauma, AAST (American Association for the Surgery of Trauma), rhabdomyolysis, hyperkalemia, anastomosis

## Abstract

Duodenal injuries are rare and difficult to diagnose, with an incidence between 1 and 5% in cases of abdominal trauma. We present the case of a 30-year-old man who suffered a motorcycle collision presented with a 24-hour history of abdominal pain, peritoneal tenderness, and hemodynamic instability. Imaging studies show evidence of free fluid in the perihepatic, perisplenic, and pelvic space. An exploratory laparotomy was performed, finding a grade III duodenal, grade V jejunal, and grade II pancreatic injuries. The basis of surgical treatment being a primary anastomosis of duodenal and jejunal injuries, which allowed discharging him home 8 days after surgery and without any complications in his follow-up.

Duodenal injuries are uncommon and difficult to diagnose, 1 with an incidence between 1 and 5% in cases of abdominal trauma. 2 3 In a review by García Santos et al, 3 which included 23 case series of duodenal injury, it was found that the ratio of penetrating to blunt abdominal trauma was 3.9:1. Among blunt abdominal trauma, the most frequent mechanism of injury was due to motor vehicle crashes in 85%, due to the crushing of the duodenum with the steering wheel and spine. 3 4 Among penetrating injuries, 81% were caused by gunshot wounds and 19% by stab wounds. 3

The mortality associated with this type of injury ranges from 18 to 30%. 1 3 Early deaths are due to massive bleeding from major vascular injuries or associated head trauma, while late deaths are associated with sepsis, duodenal fistulas, and multiorgan failure. It is imperative to recognize these types of injuries in a timely manner, since the most important risk factor associated with mortality is the delay between diagnosis and treatment, 3 given that a diagnostic delay in the first 24 hours can increase mortality up to four times. Other risk factors that increase the mortality rate include the presence of an associated pancreatic injury and injury to the common bile duct. 3 5

Since the duodenum is surrounded by other organs and vital structures, associated intra-abdominal injuries are present in 68 to 100% of cases. 1 3 4 The trauma kinematics play an important role in the severity and the organ affected, with the structures with the highest injury rate being the liver 17%, pancreas 12%, small intestine 11%, colon 13%, stomach 9%, biliary tract 6%, kidney and urinary tract 6.5%, spleen 4.1%, and vascular injuries such as aorta, vena cava, and portal vein in up to 15%. The vascular injuries pose the highest mortality rate due to the high possibility of death from massive bleeding in the first minutes to hours after the injury. 3

Regarding the duodenum, an analysis was carried out on a total of 1,042 patients where the most frequent site of injury was the second portion (D2) in 36%, followed by the third portion (D3) in 18% and the fourth portion (D1) in 15%. The least frequently injured duodenal portion was the first (D1), with 13%, and multiple portion injuries were found in 18%. 3

## Clinical Case

A 30-year-old male with no comorbidities and a positive history of methamphetamine (crystal meth) use was treated in a private institution for loss of consciousness following a motorcycle collision. No relevant abdominal findings were noted at that moment. Chest X-rays and a computed tomography (CT) scan of the head were performed, and he was discharged with no neurological abnormalities. After 48 hours, he began experiencing severe abdominal pain, signs of high intestinal obstruction, and syncope, prompting transfer to our institution.

Upon arrival, the patient had a Glasgow Coma Scale score of 13, frank pallor, marbled skin, tachycardia at 120 beats per minute, tachypnea at 40 revolutions per minute, hypotension at 90/60 mm Hg, and a fever of 38.5°C. Abdominal examination revealed absent bowel sounds, diffuse abdominal tenderness, and rigidity and tympanic percussion over the liver.

**Fig. 1:**
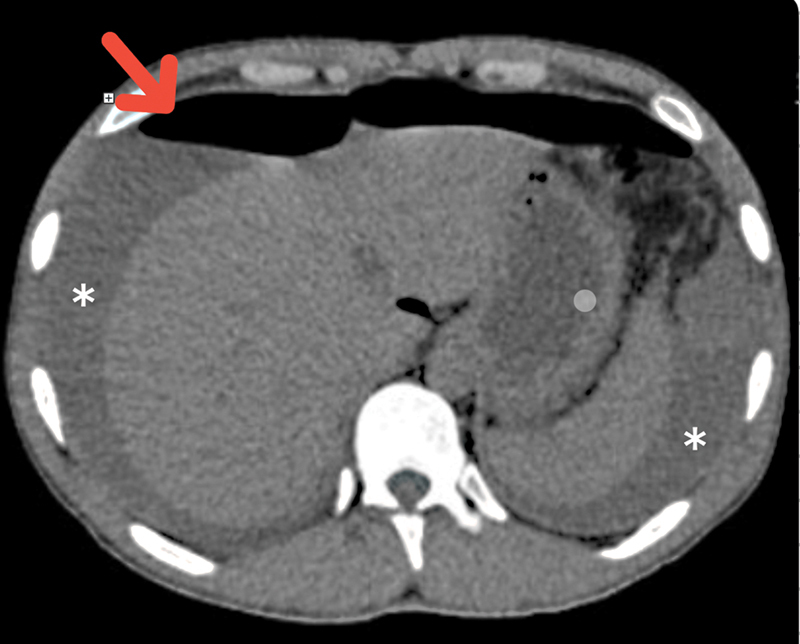
Abdominal computed tomography (CT) scan. Free intraperitoneal air, free fluid surrounding the spleen and liver (the arrow signifies the free intraperitoneal air).

**Fig. 2:**
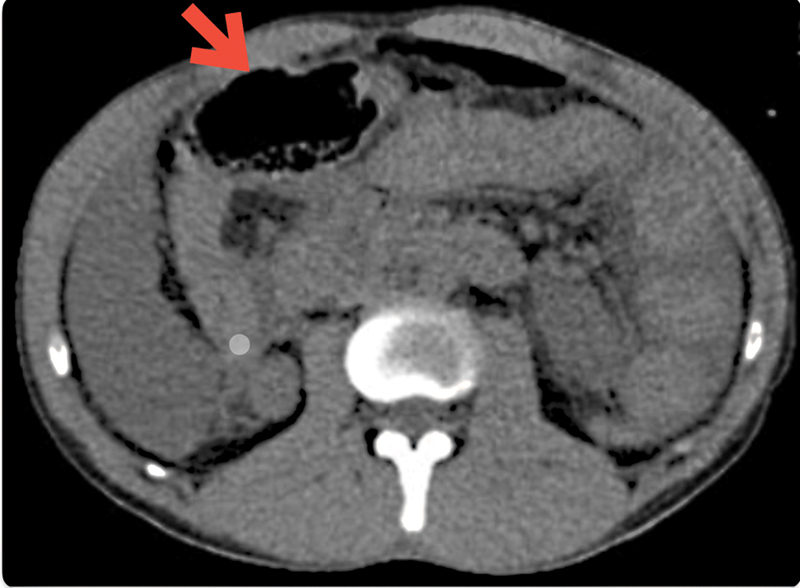
Abdominal computed tomography (CT) scan showing duodenal disruption with intestinal wall pneumatosis (the arrow signifies the duodenal disruption).

An abdominal CT scan revealed free fluid in the perihepatic, perisplenic, and pelvic spaces, and free air in the abdominal cavity, as seen in Fig. 1 . There was also evidence of possible duodenal disruption, as seen in Fig. 2 .

Prior to surgery, the patient received massive fluid resuscitation with 0.9% sodium chloride, broad-spectrum antibiotics, analgesia, nasogastric tube catheter, and urinary catheter with a urine output of 0.4 mL/kg/hour. Blood products, including packed red blood cells, plasma, and platelet concentrates, were also administered.

**Fig. 3:**
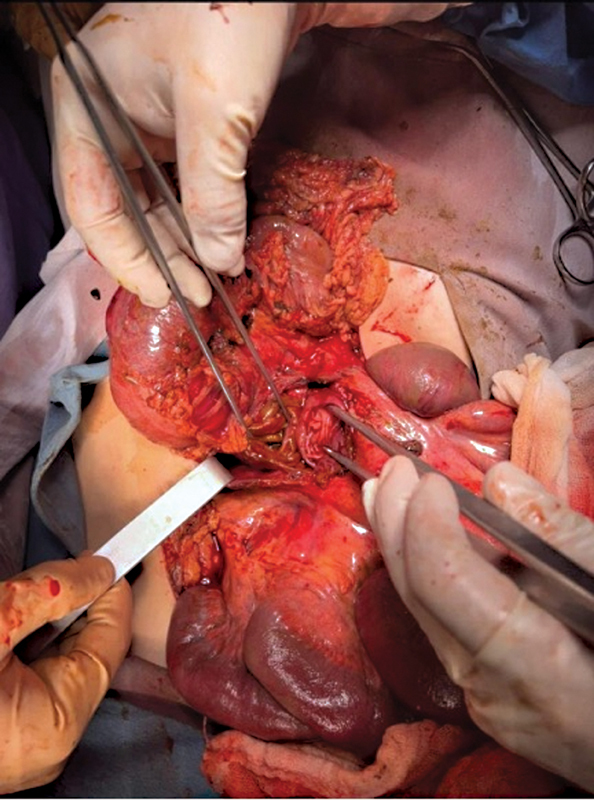
Grade III duodenal laceration of D3.

**Fig. 4:**
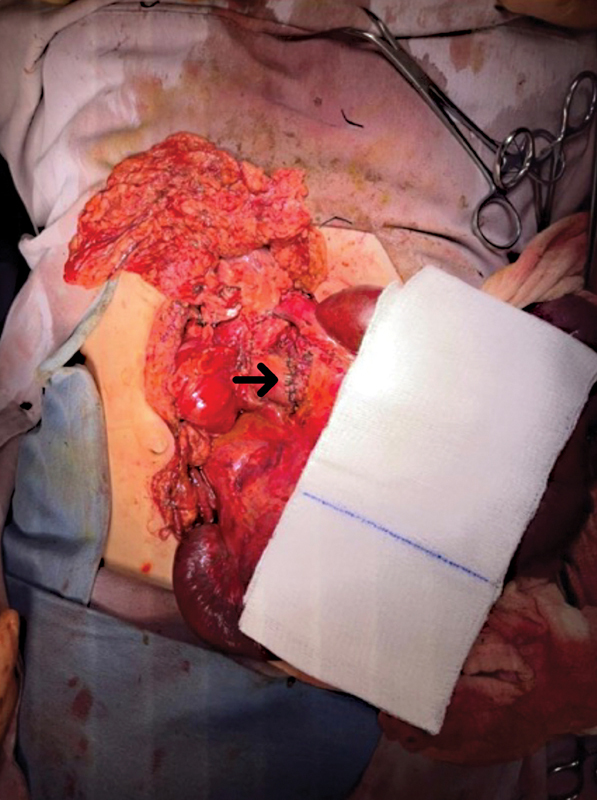
Duodenal end-to-end anastomosis (the arrow signifies the duodenal end-to-end anastomosis).

Surgical exploration via a midline laparotomy incision revealed 2,750 mL of hemobiliary fluid and several organs injured. A duodenal injury was identified in the third portion of the duodenum, with an American Association for Trauma Surgery (AAST) grade III disruption (50–100% circumferential disruption), as seen in Fig. 3 . A pancreatic injury AAST grade II, with superficial laceration and no involvement of the main ducts. One jejunal injury AAST grade V, with a 5-cm segmental loss of tissue 100 cm from the angle of Treitz. The patient also presented a vascular injury to the middle colic artery in its lateral portion, which was repaired with 4-0 Vicryl suture. The duodenal and jejunal injuries were repaired with two-layer end-to-end anastomosis without tension, as seen in Fig. 4 . The abdominal cavity was washed out with 4 L of warm solution. During the postoperative period, the patient had low urine output, metabolic acidosis, and shock, necessitating vasopressor support with norepinephrine and bicarbonate administration.

In the first 24 hours after surgery the patient developed rhabdomyolysis, worsening of the kidney function, and hyperkalemia with a potassium level of 6.9 mmol/L. He also demonstrated electrocardiographic changes consistent with supraventricular tachycardia, probably associated to the vasopressor use and meth consumption. One session of hemodialysis was sufficient to help restore kidney function. Early total parenteral nutrition was initiated, and the nasogastric tube was removed on the second day, with enteral feeding started on the fifth day without evidence of leakage via Penrose drain.

The patient was discharged without complications on the eighth day postoperatively. Three outpatient follow-up visits showed only scant serous fluid around the wound, which was drained without complications. The fluid was sent for analysis to rule out pancreatic fistula, which tested negative for amylase and lipase.

## Discussion

The diagnosis of duodenal injury can be difficult, especially in cases of blunt abdominal trauma, as the symptoms may be unspecific; they may present with abdominal tenderness and peritonitis on initial evaluation, highly suggestive of intra-abdominal injuries, but not specific to a duodenal injury. 4 5

Focused Assessment with Sonography in Trauma (FAST) is a widely accepted and useful method in cases of blunt abdominal trauma; however, it has low sensitivity for duodenal injuries, as up to 30% of patients with some type of retroperitoneal injury, including the duodenum, may have a normal FAST exam. 4 5

Abdominal radiography in the upright position may suggest duodenal injury if right psoas muscle effacement or retroperitoneal air is found, however, it is unreliable. 3

CT is one of the best methods for diagnosing duodenal injuries in hemodynamically stable patients, even without the need for hydrosoluble contrast. 1 The sensitivity of CT for detecting biliopancreato-duodenal injuries approaches 83%, decreasing to 79% for biliopancreatic (BP) injuries and 50% for bilioduodenal injuries. 5 6 The findings of a duodenal injury include thickening of the wall, periduodenal or right pararenal fluid, decreased enhancement in the injured duodenal segment, and accumulation of clots near the site of injury, which is visualized as a heterogeneous fluid collection (the “sentinel clot sign”). 4 Findings suggestive of duodenal perforation include the presence of retroperitoneal air, wall disruption, and contrast extravasation. 3 5

Magnetic resonance imaging is more sensitive than CT for detecting low-grade injuries; however, it is more expensive and lacks utility in the context of trauma; its use is generally reserved for the evaluation of associated biliary or pancreatic ductal injuries. 5

### Classification

There are different scales developed to classify the severity of injuries in the context of trauma, such as the World Society of Emergency Surgery or the AAST, the latter classifying duodenal injuries into five grades ranging from hematoma or laceration to massive disruption of the pancreatoduodenal complex 7 ( Table 1 ).

**Table 1:** AAST duodenum injury scale

Grade a	Type of injury	Injury description
I	Hematoma	Single portion fo duodenum
	Laceration	Partial thickness
II	Hematoma	More than one portion
	Laceration	< 50% of circumference
III	Laceration	50–75% of D2 circumference
		50–100% of D1, D3, or D4 circumference
IV	Laceration	> 75% of D2 circumference
		Involving ampulla or bile duct
V	Laceration	Massive disruption of duodenopancreatic complex
	Vascular	Devascularization of duodenum

Abbreviations: AAST, American Association for the Surgery of Trauma; D1, duodenum first portion; D2, duodenum second portion; D3, duodenum third portion; D4, duodenum fourth portion.

Note: Adapted from Moore et al, 1990. 7

a Advance one grade for multiple injuries up to grade III.

### Medical Treatment

The initial management of all trauma is based on the ATLS (Advance Trauma Life Support); if the patient is hemodynamically stable and demonstrates grade I or II duodenal injury without other associated intra-abdominal injuries, conservative management can be used, which consists of fasting, placement of a nasogastric tube, close monitoring, and a CT scan in 12 to 24 hours in case of clinical deterioration. In case of grade, I or II hematoma with clinical signs of intestinal obstruction, monitoring for 14 days is recommended, and if there is no resolution, surgical management should be considered. 2

### Surgical Treatment

Up to 70% of duodenal injuries can be successfully resolved by primary repair, leaving a remaining 30% that may require more complex procedures. 1 2 3 This includes pyloric exclusion, decompression, or duodenal diverticulization, among others, especially for complex injuries. 1 8 Complex injuries are those that involve 75% of the duodenal wall, the first or second portion of the duodenum, those with a repair delay greater than 24 hours, and those associated with pancreatic or bile duct injuries. 3

AAST grade I to II injuries: lacerations should be repaired in a transverse manner with imbricating stitches, without tension, and nonviable edges should be debrided. No superiority has been shown in performing closure in one or two layers. In case of a hematoma that is obstructing > 50% of the lumen, it should be carefully drained. 2

AAST grade III–V injuries that do not involve the duodenopancreatic complex can be managed in the same way as minor injuries, by debriding necrotic edges and primary closure or anastomosis. 4

AAST grade IV and V injuries involving the ampulla increase the severity and complexity of management. 4 For injuries limited to the ampulla, there is the possibility of placing stents or performing sphincteroplasty. However, in case of total avulsion of the ampulla, a choledocoduodenal anastomosis should be considered. Other extensive injuries in this area, whether intramural or intrapancreatic of the common bile duct, may be candidates for a pancreatoduodenectomy. 3 9

Any method of duodenal exclusion has shown the same rates of complications and mortality, 8 but pyloric exclusion is the most commonly used procedure because it is technically simpler. 4 Despite this, it offers little advantage over primary repair with adequate decompression using a nasogastric tube, adding operative time and increasing the risk of an extra anastomosis. 1 Another consideration for this procedure is the average time of spontaneous reopening at 3 weeks regardless of the technique used. It is a frequent site of marginal ulcers with an incidence up to 33%, so in addition to the surgical time and its few demonstrable benefits, it is a procedure that is performed every time with less frequency. 10

#### Duodenostomy

Duodenojejunostomy with creation of a Roux-en-Y anastomosis remains an accepted treatment, especially in patients in whom primary repair could cause duodenal stenosis greater than 50%, or in patients with associated pancreatic injuries. 1 With injuries involving the first or second proximal portion of the duodenum, an antrectomy and Billroth II reconstruction may be an option. 2

#### Pancreaticoduodenectomy

This complex procedure is reserved for injuries that damage the pancreatoduodenal complex, with ampullary destruction and BP ducts involvement. Other indications include massive or difficult to control bleeding. 1

In a retrospective review from the Panamerican Trauma Society which included 11 centers and 372 patients over a 10-year period, primary repair was the most common type of surgical management (80%). Sixteen patients underwent pyloric exclusion with gastrojejunostomy, 13 had pyloric exclusion without gastrojejunostomy, 37 had primary repair with retrograde decompressive duodenostomy with or without distal feeding tube, 5 had resection with primary anastomosis, and 2 had a Whipple procedure. Primary repair was used in 80% of patients, even in high-grade duodenal injuries, and 2.5% of patients had injuries where primary repair was not possible. Primary repair was a safe and effective way to treat duodenal injuries. Although there are more complex options available, it is not clear that they are better, thus, primary closure is still the treatment of choice. 8

A review by Siboni et al 11 with a total of 743 patients with duodenal trauma showed 280 (37.7%) underwent primary repair, 68 (9.2%) gastrojejunostomy, and 5 (0.7%) pancreatoduodenectomy. For most injuries, primary repair was performed regardless of the severity of the duodenal injury. Although there was no statistical difference in the choice of procedure according to the severity of the injury, in a small number of complex duodenal injuries, surgeons tended to be less likely to perform primary repair. Hospital mortality and postoperative sepsis were similar in patients undergoing primary repair versus gastrojejunostomy, even in high-grade injuries (mortality 6.6% vs. 4.5%, *p* = 0.777, sepsis: 10.4% vs. 6.7%, *p* = 0.578). The mean time of hospital stay, however, was significantly shorter in patients treated with primary repair (11 vs. 18 days), with no significant difference in sepsis or mortality rate. 11

## Complications

Duodenal injuries are associated with a complication rate up to 65%, such as fistulas (6–33%), intra-abdominal abscesses and sepsis (17.8%), or posttraumatic pancreatitis (3–15%). 2 4 8

The most important risk factor for the development of complications is the presence of preoperative and intraoperative hypotension. 10 Other risk factors include blunt abdominal trauma, high-speed projectiles, the degree of duodenal injury, delay of more than 24 hours to treatment, and the presence of bile duct injury. 4

A retrospective cohort study analyzing primary repair in duodenal injuries, compared physiological variables between patients who had leaks and those who did not; pH and lactate were the two physiological parameters most associated with this adverse outcome. 9 In addition, the percentage of leaks was classified according to the anatomical site of the affected duodenum. A total of 91 duodenal injuries were analyzed, D1 (31), D2 (12), D3 (37), and D4 (16), the chi-square test revealed a significant difference in the leak rate between AAST-I (0%), AAST-II (1.6%), and AAST-III (66.7%) injuries ( *p* < 0.01). Six out of seven (86%) patients who developed a leak had an AAST grade III injury and one had grade II injury. AAST grade III injuries also had significantly higher mortality rates (33.3%) than grade II (9.3%) and grade I (11.1%) injuries ( *p* = 0.04). 9

It is essential for the comprehensive management of patients with severe abdominal trauma to identify rhabdomyolysis as the main cause of acute renal failure. Timely management is based on rapid and effective restoration of volume status, improvement of renal blood perfusion, use of renal protectants, administration of diuretics with normal volume status, and especially in rhabdomyolysis and oliguria, control of acidosis; as well as monitoring and correction of hyperkalemia, even considering hemodialysis. 12

## Conclusion

There are several surgical techniques described for treating high-grade duodenal injuries, some more complex than others. However, primary closure has been shown to be superior in terms of postoperative results, showing to be the least complex procedure and the fastest to perform. Therefore, it can be applied in patients with this type of duodenal injury who are also hemodynamically unstable.
